# The Aftermath of Tapering Tocilizumab After Achieving Treatment Target in Patients With Rheumatoid Arthritis: A Nationwide Cohort Study

**DOI:** 10.3389/fmed.2022.839206

**Published:** 2022-02-08

**Authors:** Jun Won Park, Min Jung Kim, Hyoun-Ah Kim, Jin Hyun Kim, Eun Bong Lee, Kichul Shin

**Affiliations:** ^1^Division of Rheumatology, Department of Internal Medicine, Seoul National University College of Medicine, Seoul, South Korea; ^2^Division of Rheumatology, Department of Internal Medicine, Seoul Metropolitan Government—Seoul Boramae Medical Center, Seoul, South Korea; ^3^Department of Rheumatology, Ajou University School of Medicine, Suwon, South Korea; ^4^Division of Rheumatology, Department of Internal Medicine, Chungnam National University College of Medicine, Daejeon, South Korea

**Keywords:** rheumatoid arthritis, tocilizumab, dose tapering, treat-to-target, bDMARD therapy

## Abstract

**Background:**

Although recent guidelines recommend that tapering of biologic disease-modifying anti-rheumatic drugs (bDMARDs) can be considered in patients with rheumatoid arthritis (RA), there has been little evidence supporting the strategy during the non-tumor necrosis factor inhibitor treatment. This study aims to investigate the effectiveness and safety of tapering tocilizumab (TCZ) dose in patients with RA who attain low disease activity (LDA) after TCZ therapy in a nationwide cohort.

**Methods:**

Data were collected from a nationwide cohort of patients with RA receiving biologic disease-modifying anti-rheumatic drugs in South Korea (KOBIO-RA). This study included 350 patients who were treated with TCZ and achieved Clinical Disease Activity Index-low disease activity (CDAI)-LDA (CDAI ≤ 10) after 1 year of treatment. We performed longitudinal analysis considering clinical data measured at all 1-year intervals for the included patients using the generalized estimating equation. A total of 575 intervals were classified into two groups according to their dose quotient (DQ) of TCZ (tapering group vs. standard-dose group). The main outcome was maintaining CDAI-LDA in the following 1-year interval.

**Results:**

Tapering TCZ dose strategy was used in 282 (49.0%) intervals with a mean (SD) DQ of 66.0 (15.5) %. Loss of CDAI-LDA occurred in 91 (15.1%) intervals. Multivariable GEE showed that the tapering group was associated with more frequent failure to sustain CDAI-LDA (adjusted OR [95% CI]: 0.57 [0.33–0.99]), which subsequently led to impaired functional status. The likelihood of achieving DAS28-deep remission (DAS28-ESR <1.98) was also significantly lower in the tapering group (adjusted OR 0.68 [0.46–0.99]). CDAI remission was achieved in only 69 (12.0%) of the total intervals, with no significant difference in the proportion of intervals achieving the target between the two groups. Incidence of adverse events was comparable in both groups except for hypercholesterolemia, which was lower in the tapering group.

**Conclusions:**

Tapering TCZ dose after achieving LDA increases the risk of losing LDA without a significant merit in safety.

## Introduction

The treatment paradigm of rheumatoid arthritis (RA) has been altered by the introduction of biologic disease-modifying anti-rheumatic drugs (bDMARDs). An increased number of treatment options has enabled rheumatologists to implement a “treat-to-target” (T2T) strategy to attain the optimal treatment target (remission or low disease activity, LDA) (1). Although bDMARDs are the cornerstone of the T2T strategy (2–4), long-term use of bDMARDs can increase the risk of severe adverse events and be an economic burden. This suggests that the optimal strategy for bDMARD therapy after achieving the treatment target remains unclear (5, 6).

In order to answer this, there have been many randomized controlled trials (RCTs) that investigated the efficacy of tapering bDMARD dose, mainly tumor necrosis factor inhibitors (TNFi), in patients who attained the treatment goal (7–9). They showed that a tapering strategy had comparable efficacy and safety to continued standard-dose bDMARD treatment in patients who achieved remission or LDA. Based on these results, recent guidelines from the American College of Rheumatology (ACR) and European League Against Rheumatism (EULAR) recommend that tapering bDMARD dose can be considered in patients with sustained (at least 6 months) remission or LDA (10, 11). However, there are some limitations to the application of this strategy in the real world. First, many cohort studies of patients with RA showed that achieving sustained remission is uncommon, especially in patients with a long disease duration (2, 12, 13). Second, treatment protocols for tapering bDMARD dose are fairly heterogeneous in the literature (14). Finally, only a few studies have investigated the effectiveness and safety of tapering bDMARD dose compared with standard treatment using non-TNFi bDMARDs (15). These led to different opinions among rheumatologists regarding the timing and type of patients who would benefit from tapering bDMARD dosage, especially non-TNFi agents (16).

Tocilizumab (TCZ), a recombinant humanized monoclonal antibody against the human interleukin-6 (IL-6) receptor, is the most commonly prescribed bDMARD for the treatment of RA in South Korea (17). As tapering TCZ dose in patients with RA is not an uncommon practice in South Korea, longitudinal follow-up data of these patients can provide evidence regarding its impact on the patients' outcomes. Here, we aimed to investigate the efficacy and safety of tapering TCZ dose in patients with RA who attained LDA after TCZ therapy using data from a nationwide cohort of bDMARD users with RA.

## Methods

### Study Population

Data were collected from the Korean College of Rheumatology Biologics Registry (KOBIO-RA) cohort, a nationwide cohort of patients with RA receiving bDMARDs in outpatient clinics (NCT01965132). From December 2012 to January 2020, the cohort enrolled 2,272 patients from 47 referral hospitals in South Korea. All patients fulfilled the ACR/EULAR 2010 classification criteria at the time of diagnosis. Patients were enrolled when they initiated a new bDMARD therapy and were followed-up annually thereafter. Of the total subjects, 687 patients received TCZ treatment. We selected patients treated with TCZ for more than 1 year and achieved Clinical Disease Activity Index (CDAI) LDA (CDAI ≤ 10) at the 1-year follow-up. Patients who missed follow-up visits, and those with missing CDAI data or TCZ doses were excluded. Patients starting with a lower dose (<8 mg/kg once every 4 weeks for IV administration and <162 mg once every other week for subcutaneous (SC) administration) of TCZ were also excluded. Finally, a total of 350 patients were analyzed in this study. A schematic of the inclusion/exclusion process is presented in Supplementary Figure 1.

This study was carried out in accordance with the Declaration of Helsinki and was approved by the institutional review board (IRB) of the coordinating center (Seoul Metropolitan Government—Seoul Boramae Medical Centre, Seoul, Korea [IRB No. 26-2012-34]) and then by each participating referral center. Written informed consent was obtained from all the patients.

### Data Collection

The baseline visit was the date of starting TCZ treatment. Data regarding demographics, smoking status (current-/ex-/never-smoker), body mass index (BMI), comorbidities, and previous bDMARD use (yes or no) were collected at baseline. Data on swollen/tender joint counts (0–44), patient and physician visual analog scale (VAS), serum levels of acute phase reactants (erythrocyte sedimentation rate (ESR) and C-reactive protein (CRP), functional status, and concomitant medications were longitudinally collected at every follow-up visit. Disease activity was estimated using the following indices: Disease Activity Score-28 for RA with ESR (DAS28-ESR), CDAI, and Simplified Disease Activity Index (SDAI). Functional status was estimated using the Health Assessment Questionnaire Disability Index (HAQ-DI), which was converted from Multidimensional Health Assessment Questionnaire (MDHAQ) using a validated formula (18, 19). Severe functional disability was defined when a patient's HAQ-DI score was >2. Remission was defined as DAS28-ESR (<2.6), CDAI ( ≤ 2.8), SDAI ( ≤ 3.3), and ACR/EULAR Boolean definitions (20). The safety of TCZ was assessed at the proportion of 1-year intervals in which any specific adverse event (AE) occurred. Each AE was graded based on the Common Terminology Criteria for Adverse Events (CTCAE) v5.0, and those with ≥ grade 3 AEs were considered to be having severe AEs. For the safety assessment, data from the following patients were also used for the analysis: (1) those who failed to achieve CDAI-LDA at 1-year follow-up (*n* = 197); (2) those without 1-year CDAI (*n* = 4); and (3) those who were initially treated with a lower dose of TCZ (*n* = 28).

The dose and interim of TCZ therapy in each one-year interval was converted to a dose quotient (DQ), calculated as (mean actual dose/standard dose) × (standard – dosing interval/mean actual dosing interval) × 100 (21, 22). Each 1-year interval was classified into the control (DQ = 100) and tapered group (DQ <100) according to the DQ of the interval. The observation period in this study was 4 years from the baseline visit or discontinuation of TCZ, whichever came first.

### Tapering of Tocilizumab and Outcomes

The decision on dose or interim adjustment of TCZ was mainly based on a shared decision between the patient and the treating rheumatologist. In our data, annual DQ for each patient changed continuously during the observation period (Supplementary Figure 2) and was guided by the disease activity of the previous visit (*P*-value for type III test <0.001). Therefore, we performed a longitudinal analysis to consider the effect of all clinical variables measured in each one-year interval on the change in disease activity over time. Besides TCZ, dose adjustment of concomitant medication such as methotrexate (MTX) or glucocorticoids was at the discretion of the attending physician.

The main outcome of this study was maintaining CDAI-LDA in a 1-year interval. Secondary outcomes included achieving remission of: CDAI (CDAI ≤ 2.8), DAS28 (DAS28-ESR <2.6), SDAI (SDAI ≤ 3.3), DAS28 deep remission (DAS28-ESR <1.98), and ACR/EULAR Boolean definition of remission, and proportion of 1-year intervals in which a specific AE occurred (23).

### Statistical Analysis

All statistical analyses were based on observational data without imputation of missing values because the proportion of missing data for all variables used in this study was <1%. The effect of tapering TCZ dose was estimated using generalized estimating equation (GEE) models. GEE is a suitable technique to make use of all available clinical data measured repeatedly in each patient and to allow for specification of both time-varying variables and individual differences (24). An “autoregressive 1” correlation matrix was selected based on the best fit of the data. The final multivariable model included clinical factors with relevant associations (*P* < 0.2) with outcomes in the univariable model.

As a sensitivity analysis, the effect of tapering TCZ dose was estimated in the multivariable GEE model, including all clinically relevant variables *a priori*. In addition, we applied “exchangeable” correlation matrix in the same GEE model to investigate the effect of tapering TCZ dose on the main outcome. All statistical analyses were performed using SPSS (version 20.0; IBM Corp., Armonk, NY, USA).

## Results

### Patient Characteristics

The baseline characteristics of the included patients are presented in Table 1. The mean (SD) age was 53.9 (13.0) years, 83.7% patients were female, and the mean disease duration was 8.2 (7.8) years. The mean BMI was 22.5 (3.3) and 19.1% of patients were obese (BMI ≥ 25). A total of 261 (74.6%) patients were bDMARD-naïve. Before initiating TCZ therapy, 286 (81.7%) patients had received methotrexate, and most patients (*n* = 322, 92.0%) had been treated with at least one conventional synthetic DMARD (csDMARD) treatment. Oral glucocorticoids were administered to 311 (88.9%) patients at baseline.

**Table 1:** Baseline characteristics of the patients.

**Clinical features (*N* = 350)**	
Age, year, mean (SD)	53.9 (13.0)
Female sex, *n* (%)	293 (83.7)
Body weight, kg, mean (SD)	57.1 (9.8)
BMI, mean (SD)	22.5 (3.3)
Obesity (BMI ≥ 25)	67 (19.1)
Smoking status	
Never smoker, *n* (%)	301 (86.0)
Ex-smoker, *n* (%)	33 (9.4)
Current smoker, *n* (%)	16 (4.6)
Number of comorbidities, mean (SD)	0.7 (0.9)
Disease duration, year, mean (SD),	8.2 (7.8)
Seropositive RA, *n* (%)	326 (93.1)
TCZ as the first use of bDMARD, *n* (%)	261 (74.6)
Tocilizumab SC agent, *n* (%)	69 (19.7)
Swollen joint count (0-44), mean (SD)	6.0 (5.2)
Tender joint count (0-44), mean (SD)	7.3 (6.1)
ESR, mm/hr, mean (SD), (n = 348)	50.5 (28.5)
CRP, mg/dL, mean (SD), (n = 348)	2.5 (3.2)
Patient global assessment (0-10), mean (SD)	6.9 (2.1)
Physician global assessment (0-10), mean (SD)	6.3 (2.1)
DAS28-ESR, mean (SD), (n = 348)	5.44 (1.25)
CDAI, mean (SD)	25.5 (11.2)
SDAI, mean (SD), (n = 348)	28.0 (12.0)
HAQ (0-3), mean (SD)	1.3 (0.8)
Number of csDMARDs before starting TCZ, mean (SD)	1.3 (0.7)
Glucocorticoids before starting TCZ, *n* (%)	311 (88.9)

*bDMARD, biologic disease-modifying anti-rheumatic drug; BMI, body mass index; CCP, cyclic citrullinated peptide; CDAI, clinical disease activity index; CRP, C-reactive protein; csDMARD, conventional synthetic disease-modifying anti-rheumatic drug; DAS, disease activity score; ESR, erythrocyte sedimentation rate; HAQ, health assessment questionnaire; RA, rheumatoid arthritis; SC, subcutaneous; SD, standard deviation; SDAI, simplified disease activity index; TCZ, tocilizumab*.

At the baseline visit, the mean (SD) CDAI was 25.5 (11.2), and all patients showed CDAI moderate/high disease activity. The mean HAQ was 1.3 (0.8), and 73 (20.9%) patients showed severe functional disability. After 1 year of treatment, CDAI significantly reduced to 5.8 (2.7). However, CDAI remission was achieved in only 54 patients (15.4%). The mean HAQ at the 1-year follow-up visit was 0.7 (0.5), with 6 (1.7%) patients having severe disability (Supplementary Table 1).

### Longitudinal Follow-Up of Disease Activity

Among the 575 1-year intervals, CDAI-LDA was maintained at 484 (84.9%) intervals. The proportion did not change according to the follow-up year (*P*-value for trend = 0.313). DAS28-based remission and deep remission were achieved in 416 (73.0%) and 299 (52.5%) intervals, respectively. In contrast, remission defined according to the CDAI, SDAI, and ACR/EULAR-Boolean criteria was attained only in 69 (12.0%), 104 (18.1%), and 99 (17.4%) intervals, respectively (Supplementary Figure 3).

During the observation period, TCZ discontinuation occurred in 56 (9.7%) intervals, and its frequency was comparable between the standard-dose and the tapering group (9.9 vs. 9.6%, *P* = 0.896).

### Clinical Factors Associated With Efficacy Outcomes

In the univariable GEE model, high BMI, current smoking status, SC administration, high ESR at baseline, use of concomitant glucocorticoid, and high CDAI in the previous visit were significantly associated with an increased risk of loss of CDAI-LDA. In contrast, the bDMARD-naïve status before tocilizumab treatment lowered the risk of loss of CDAI-LDA. In the multivariable GEE model, bDMARD-naïve status, subcutaneous administration, glucocorticoid use, and high CDAI in the previous visit significantly influenced on the likelihood of loss of CDAI-LDA (Table 2).

**Table 2:** Clinical factors associated with the loss of CDAI-low disease activity (LDA).

	**Univariable analysis**		Multivariable analysis^*^	
**Clinical factors**	**OR (95% CI)**	***P***	**OR (95% CI)**	***P***
Age, year	1.01 (0.99 to 1.03)	0.472	^†^	
Female sex	0.83 (0.42 to 1.64)	0.592	^†^	
Disease duration, year	1.02 (0.99 to 1.05)	0.215	^†^	
BMI	1.09 (1.002 to 1.18)	0.046	1.10 (1.01 to 1.20)	0.025
Smoking history (vs. never-smoker)		0.057	^†^	0.366
Ex-smoker	0.87 (0.34 to 2.25)		1.43 (0.51 to 4.10)	
Current-smoker	3.32 (1.22 to 9.09)		2.12 (0.69 to 6.48)	
Seropositive RA (vs. seronegative RA)	1.33 (0.54 to 3.26)	0.532	^†^	
bDMARD-naïve	0.56 (0.32 to 0.97)	0.039	0.53 (0.30 to 0.93)	0.026
SC tocilizumab (vs. IV tocilizumab)	2.03 (1.19 to 3.49)	0.010	2.36 (1.23 to 4.52)	0.010
Baseline CDAI	1.01 (0.98 to 1.03)	0.646	^†^	
Baseline ESR (mm/hr)	1.01 (1.001 to 1.02)	0.036	1.01 (0.999 to 1.02)	0.089
Baseline CRP (mg/dL)	1.04 (0.97 to 1.11)	0.250	^†^	
Baseline HAQ	1.39 (1.01 to 1.91)	0.043	1.11 (0.78 to 1.60)	0.581
Concomitant MTX use	1.15 (0.67 to 2.00)	0.614	^†^	
Concomitant steroid use	2.18 (1.28 to 3.70)	0.004	1.95 (1.14 to 3.34)	0.015
CDAI measured at previous visit	1.16 (1.10 to 1.24)	<0.001	1.10 (1.03 to 1.18)	0.003

*bDMARD, biologic disease-modifying anti-rheumatic drug; BMI, body mass index; CDAI, clinical disease activity index; CI, confidence interval; CRP, C-reactive protein; ESR, erythrocyte sedimentation rate; HAQ, health assessment questionnaire; HR, hazard ratio; IV, intravenous; MTX, methotrexate; RA, rheumatoid arthritis; SC, subcutaneous*.

* *Including covariates with relevant association (P < 0.2) with the outcome in the univariable analysis*.

† *Was not included in the multivariable model*.

As for other efficacy outcomes, age at baseline and disease duration were the only baseline factors predicting CDAI remission during the 1-year interval. Concomitant glucocorticoid use during an interval was associated with a lower likelihood of achieving CDAI- and DAS28-based remission. High disease activity measured in the previous follow-up visit significantly decreased the probability of fulfilling any remission criterion (Supplementary Tables 2–6).

### Effect of Tapering Tocilizumab Dose on Longitudinal Disease Activity

**Figure 1:**
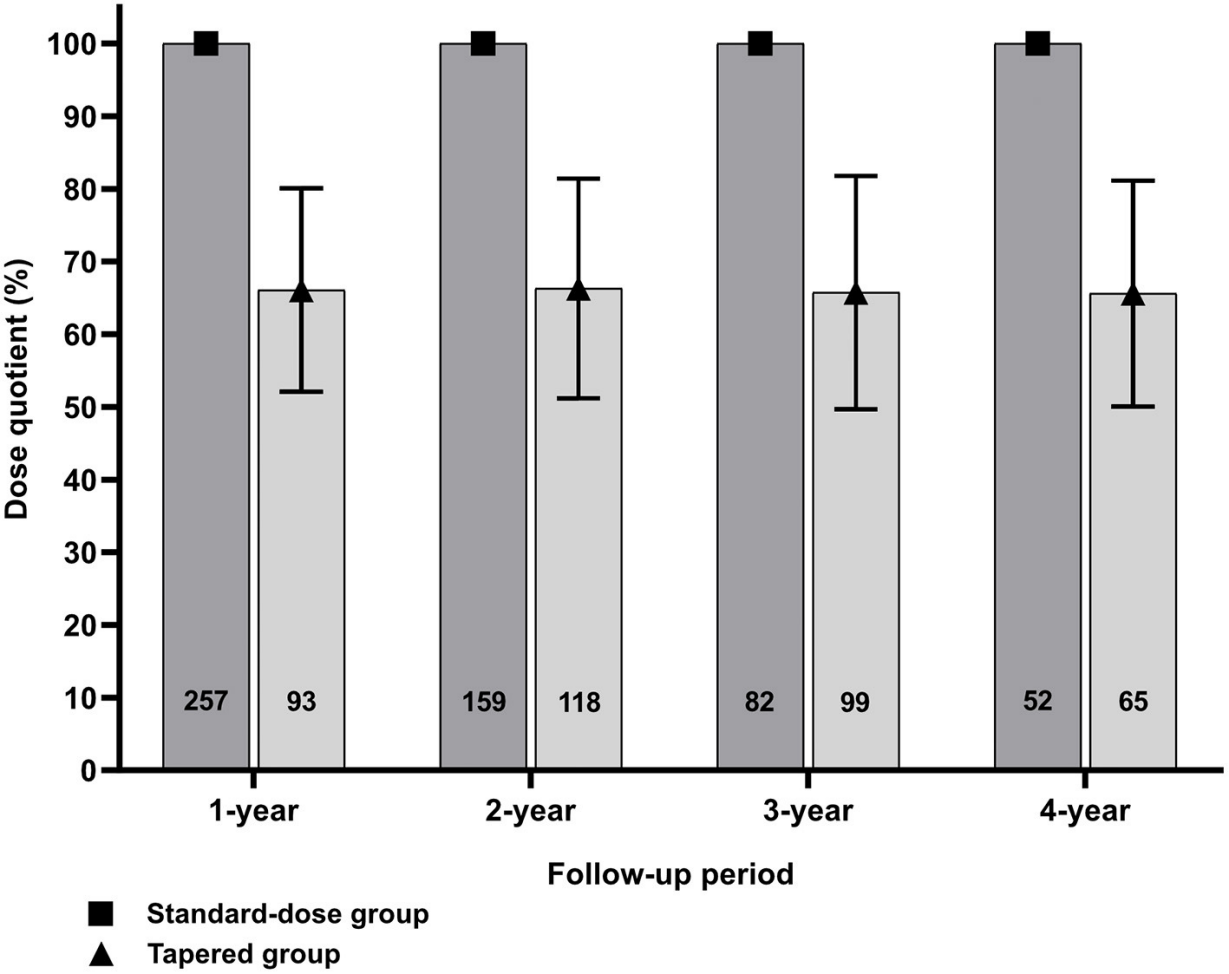
Change in dose quotient (DQ) and the number of patients over time between the two groups.

Overall, TCZ dose was tapered at 282 (49.0%) intervals. The mean (SD) DQ in the tapering group was 66.0 (15.5) and did not significantly change over time. However, the proportion of the tapering group increased over time (Figure 1). The proportion of intervals that underwent loss of CDAI-LDA was 15.3% in the tapering group and 14.9% in the standard-dose group, which was not significantly different. However, in the multivariable model, the tapering group was significantly associated with the failure to sustain CDAI-LDA (adjusted OR 0.57 [0.33–0.99]). In addition, the likelihood of achieving DAS28-deep remission was also significantly lower in the tapering group (adjusted OR 0.68 [0.46–0.99]. There was no significant difference in the frequency of attaining other remission criteria between the two groups (Table 3).

**Table 3:** Effect of tapering tocilizumab on achieving outcomes.

	**CDAI-LDA**	**DAS28-remission**	**DAS28-deep remission**	**CDAI-remission**	**SDAI-remission**	**ACR/EULAR remission**
Unadjusted OR (95% CI) (vs. standard-dose group)	0.98 (0.65 to 1.49)	1.03 (0.71 to 1.49)	0.76 (0.54 to 1.06)	1.23 (0.75 to 2.00)	0.92 (0.59 to 1.44)	0.98 (0.65 to 1.48)
Adjusted OR (95% CI) (vs. standard-dose group)	0.57 (0.33 to 0.99)	0.87 (0.54 to 1.38)	0.68 (0.46 to 0.99)	0.94 (0.57 to 1.55)	0.87 (0.54 to 1.38)	0.76 (0.50 to 1.18)

*CDAI, clinical disease activity index; CI, confidence interval; DAS, disease activity score; LDA, low disease activity; OR, odds ratio; SDAI, simplified disease activity index*.

**Figure 2:**
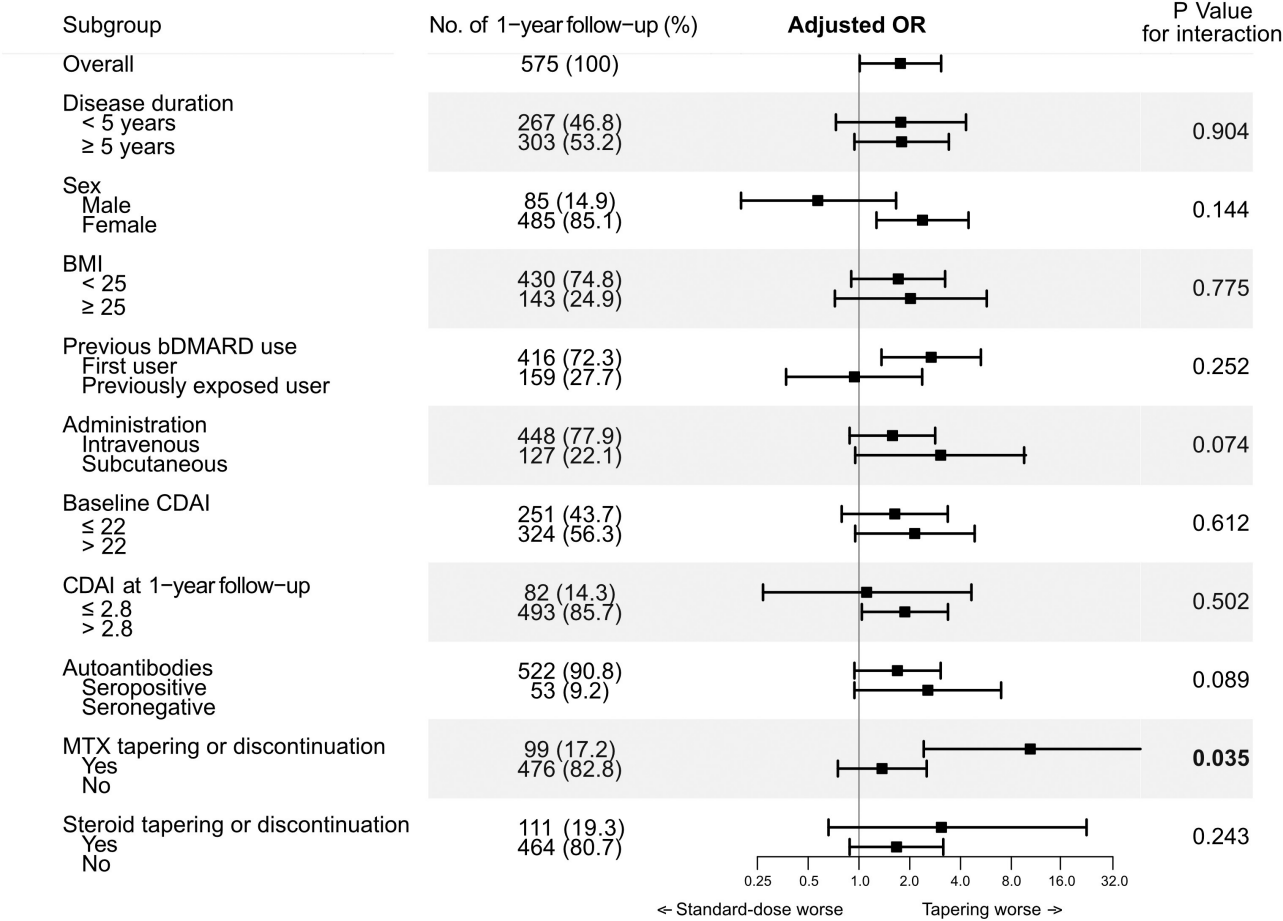
Interaction between tapering tocilizumab and clinical factors on likelihood of maintaining CDAI-LDA.

To further evaluate if the effect of tapering TCZ dose on loss of CDAI-LDA could differ according to the clinical features of the patients, the main analysis was stratified by sex, disease duration, BMI, bDMARD exposure, route of administration, baseline disease activity, autoantibody status, and concomitant tapering of MTX or glucocorticoids (Figure 2). In the subgroup of 1-year intervals in which concomitant MTX was tapered or discontinued (*n* = 99), tapering the dose of TCZ was significantly associated with loss of CDAI-LDA (adjusted OR 10.61 [2.42–46.59]). However, in the subgroup of 1-year intervals without MTX tapering or discontinuation, the association between TCZ tapering and the loss of CDAI-LDA was insignificant (adjusted OR 1.37 [0.75–2.52]).

Longitudinal changes in HAQ-DI estimated using the multivariable GEE models were comparable between the two groups (Supplementary Figure 5). However, the intervals that underwent loss of CDAI-LDA showed significantly higher HAQ-DI scores than those in which LDA was maintained or improved (Supplementary Figure 4). In addition, loss of CDAI-LDA was associated with higher CDAI in the next follow-up interval (adjusted β 2.81 [0.04–5.59]).

### Safety Assessment

Among the 1,436 intervals for the safety assessment, 857 Aes occurred in 658 (45.8%) intervals. The severity of most Aes was mild to moderate, and 21 (2.5%) were severe. Hypercholesterolemia was the most commonly occurring AE (*n* = 409), followed by elevated aspartate transaminase and/or alanine transaminase levels (*n* = 195). Bacterial infections occurred in 45 (3.1%) intervals (Table 4). The frequency of each AE was comparable between the two groups, except for fewer cases of hypercholesterolemia in the tapering group (32.8 vs. 37.9%, *P* = 0.048).

**Table 4:** Safety assessment of tapering tocilizumab compared to its standard regimen.

**Adverse event (AE)**	**Standard-dose group**	**Tapering group**	***P* value**
	**(n = 858)**	**(n = 578)**	
Any mild/moderate AE, *n* (%)			
Neutropenia	11 (1.3)	8 (1.4)	0.979
LFT abnormality (n = 1412)	125 (14.8)	69 (12.2)	0.297
TB	0 (0.0)	0 (0.0)	NA
NTM	1 (0.1)	4 (0.7)	0.110
Bacterial infection	20 (2.3)	12 (2.1)	0.808
Herpes zoster	11 (1.3)	8 (1.4)	0.883
HBV reactivation	1 (0.1)	3 (0.5)	0.093
Nasopharyngitis	26 (3.0)	14 (2.4)	0.988
Interstitial lung disease	10 (1.2)	6 (1.0)	0.195
Hypercholesterolemia (n = 1145)	250 (37.9)	159 (32.8)	0.048
Skin rash	22 (2.6)	11 (1.9)	0.400
Infusion reaction	13 (1.5)	5 (0.9)	0.280
Any serious AE, *n* (%)			
Increased AST/ALT	3 (0.4)	1 (0.2)	0.544
Bacterial infection	8 (0.9)	5 (0.9)	0.894
Herpes zoster	2 (0.2)	0 (0.0)	NA
Skin rash	0 (0.0)	1 (0.2)	NA
Infusion reaction	0 (0.0)	1 (0.2)	NA

*ALT, alanine transaminase; AST, aspartate transaminase; HBV, hepatitis B virus; NA, non-applicable; NTM, non-tuberculosis mycobacterium; TB, tuberculosis*.

### Sensitivity Analysis

Our results were consistent across all sensitivity analyses, for instance, when all clinically relevant factors were included a priori (Supplementary Table 7) and after applying “exchangeable” correlation structure in the GEE model (Supplementary Table 8).

## Discussion

Although there have been many RCTs regarding dose tapering strategies of bDMARDs in patients with RA, there is no general consensus regarding who is suitable for tapering and how to taper the dose (14). The effects of concomitant medications and co-morbidities on treatment decisions and outcomes could be different between RCTs and real-world observational studies. Therefore, the efficacy and safety of tapering bDMARDs in daily practice may differ from those reported in RCTs. Furthermore, there has been relatively scarce evidence regarding efficacy and safety of tapering non-TNFi bDMARD. In this context, the results of this study provide important evidence for clinical decisions on tapering TCZ dose in daily practice. A few previous studies suggested that tapering TCZ could be a feasible option, but they were single-center studies and included relatively a small number of patients (25, 26). To the best of our knowledge, this study is the largest cohort study that investigated the efficacy of tapering TCZ in a large nationwide cohort, which strengthens the generalizability of the results. To fully consider the heterogeneity of tapering strategies in the real world, we performed a longitudinal analysis including all clinical variables, including the dose of TCZ and disease activity measured at all 1-year intervals for the study subjects.

Our results showed that CDAI remission was achieved in ~15% of the study population, and this did not increase over time. Although previous studies have demonstrated that achieving “remission” can predict the best prognosis in patients with RA, many observational cohort studies have shown that patients do not often reach the optimal target, especially those with established RA (12, 13, 27, 28). Our results also showed that disease duration was the only baseline factor associated with achieving CDAI remission. Considering the long disease duration in this study (mean 8.2 years), CDAI-LDA could be an alternative yet reasonable goal for our study subjects (10). Interestingly, DAS28-remission was attained in more than 70% of intervals, suggesting that the definition of “remission” is heterogeneous in patients receiving TCZ (29). However, previous studies have also shown that DAS28 is unsuitable for assessing disease activity in patients on TCZ therapy due to the high weight on acute phase reactants for its scoring (30, 31).

In this study, TCZ dose tapering was performed in approximately half of the 1-year intervals after patients achieved CDAI-LDA. However, tapering the TCZ dose significantly increased the risk of failure to sustain the target at follow-up when compared with maintaining the standard-dose regimen. Furthermore, the dose tapering strategy did not reduce any adverse drug reactions, except for a slight decrease in the prevalence of hypercholesterolemia. We also showed that loss of CDAI-LDA at 1-year intervals was associated with higher HAQ-DI scores and poor disease control thereafter, which is in line with previous studies (32, 33). Although tapering TCZ dose may reduce direct medical costs, higher disease activity and impaired physical function would increase the indirect cost related to work disability (34, 35). Hence, our results suggest that the benefit of tapering TCZ dose does not overweigh the potential negative outcomes.

However, although tapering TCZ in overall showed lower efficacy than standard-dose treatment, it is noteworthy that ~85% of 1-year intervals in the tapering group maintained the target. This suggests that tapering strategy could be an effective option in certain subgroup of the patients. Interestingly, we showed that tapering or discontinuing concomitant MTX alongside tapering TCZ was associated with failure to maintain CDAI-LDA. On the contrary, subgroup analysis in the 1-year intervals without tapering or discontinuation of MTX showed comparable odds for CDAI-LDA between the two groups. This suggests that tapering strategy could be appropriate unless dose of concomitant MTX is simultaneously tapered. A previous study including RA patients who achieved sustained LDA with MTX plus TCZ also showed that patients receiving reduced dose of TCZ were at high risk of disease flare after withdrawing MTX, which supports our results (36). Unfortunately, we were not able to investigate the efficacy of tapering TCZ dose after achieving CDAI remission due to the small number of intervals achieving the target after 1 year of standard-dose TCZ treatment (*n* = 54). Nevertheless, the low rate of CDAI remission raises the question of whether tapering TCZ dose after achieving sustained remission would be the proper strategy in real-world clinical practice.

This study has some limitations. First, as patients in the KOBIO-RA cohort were followed up annually, the disease activity measured may not represent the disease activity throughout the year. However, this study only included patients who were treated with TCZ for more than 1 year and achieved CDAI-LDA at the 1-year follow-up visit. As discontinuation of bDMARDs due to inefficacy usually occurs during the first year of treatment, it is less likely that the disease activity had fluctuated before the time point of the patient's follow-up visit (37, 38). Moreover, all patients receiving bDMARD treatment in South Korea were evaluated every 6 months to determine whether they fulfilled the EULAR response criteria (39). Second, since this was an observational study, the effect of tapering TCZ dose could be biased due to “confounding by indication.” Although we performed a longitudinal analysis to consider all clinical variables and their changes throughout follow-up, some unmeasured confounders such as patient's and physician's preferences could have influenced the results. Furthermore, the decision of tapering TCZ by the treating physician could be influenced by uncaptured adverse events. Finally, we were not able to investigate the effect of tapering TCZ dose on radiographic progression due to the scope of this registry.

In conclusion, our study showed that tapering TCZ dose in patients with RA who achieved CDAI-LDA at 1-year of TCZ treatment increased the risk of losing LDA and subsequently led to functional impairment. Our results indicate that TCZ dose should be tapered in patients with LDA with caution in daily clinical practice.

## Data Availability Statement

The original contributions presented in the study are included in the article/Supplementary Material, further inquiries can be directed to the corresponding author/s.

## Ethics Statement

The studies involving human participants were reviewed and approved by Institutional review board (IRB) of the coordinating center (Seoul Metropolitan Government—Seoul Boramae Medical Center). The patients/participants provided their written informed consent to participate in this study.

## Author Contributions

KS had full access to all of the data in the study and takes responsibility for the integrity of the data and the accuracy of the data analysis. KS and JP contributed to the study concept and design. JP, MK, H-AK, JK, EL, and KS contributed to the acquisition, analysis, or interpretation of the data, and contributed to the critical revision of the manuscript for important intellectual content. JP drafted the manuscript and contributed to the statistical analysis. All authors read and approved the final manuscript.

## Conflict of Interest

The authors declare that the research was conducted in the absence of any commercial or financial relationships that could be construed as a potential conflict of interest.

## Publisher's Note

All claims expressed in this article are solely those of the authors and do not necessarily represent those of their affiliated organizations, or those of the publisher, the editors and the reviewers. Any product that may be evaluated in this article, or claim that may be made by its manufacturer, is not guaranteed or endorsed by the publisher.
