# Supplementary material for: The Aftermath of Tapering Tocilizumab After Achieving Treatment Target in Patients With Rheumatoid Arthritis: A Nationwide Cohort Study
Source: Front Med (Lausanne). 2022 Feb 8;9:839206. doi: 10.3389/fmed.2022.839206 (PMC8860908; doi:10.3389/fmed.2022.839206)
Supplement: Supplementary file 1 [file Data_Sheet_1.docx]

**Supplementary Figure 1.** Flow of the inclusion


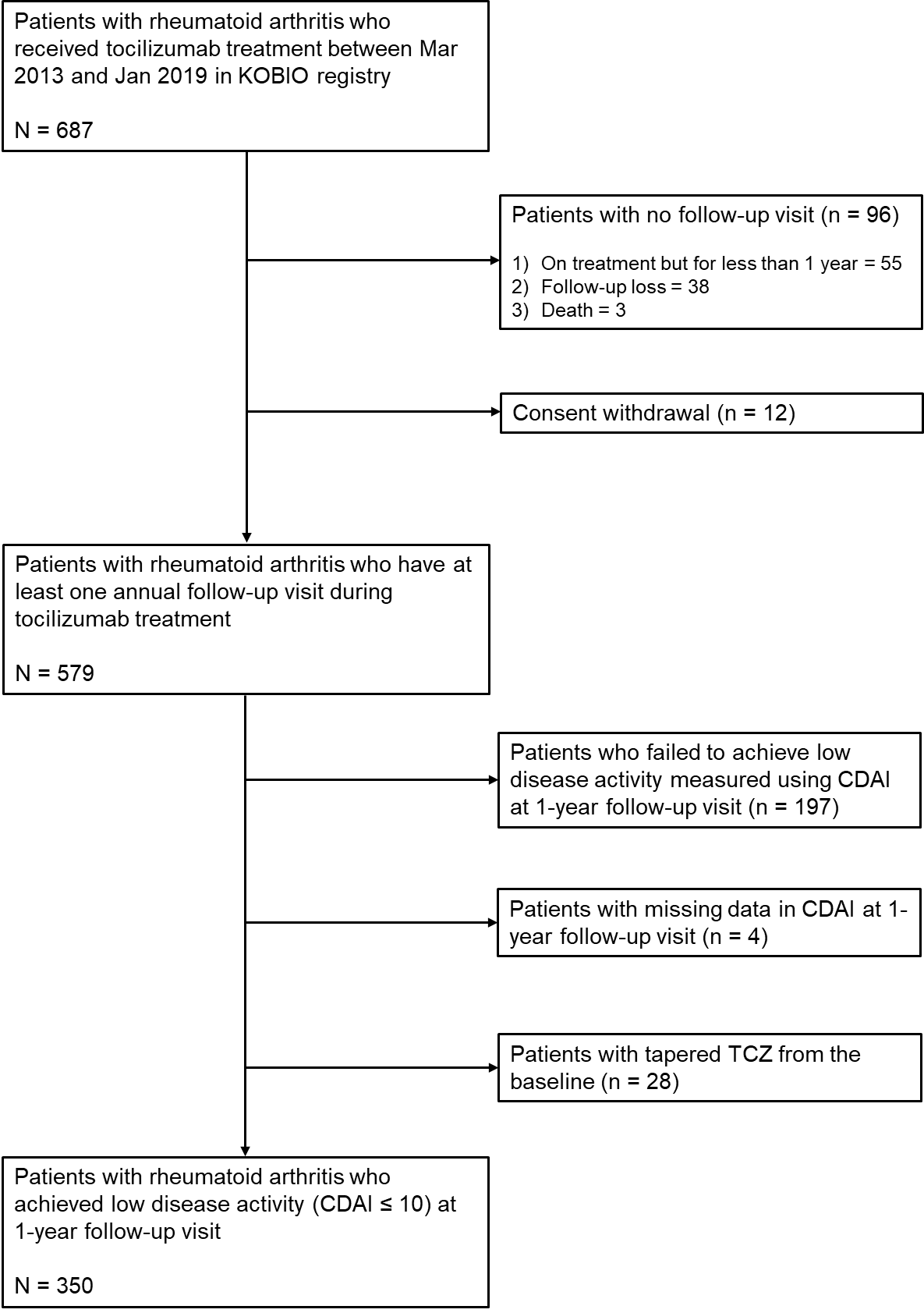


**Supplementary Figure 2.** Change in dose quotient (DQ) of tocilizumab over time in study population


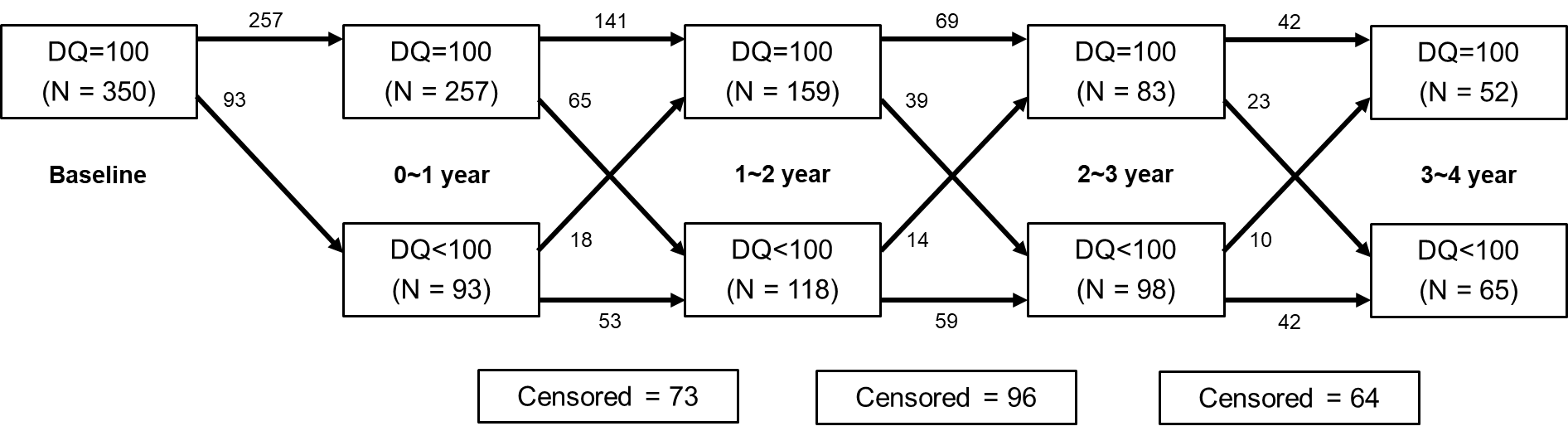


**Supplementary Table 1.** Disease activity of the study population after 1 year of tocilizumab treatment

| SJC (0-44), mean (SD) | 0.4 (0.9) |
| --- | --- |
| TJC (0-44), mean (SD) | 0.6 (1.0) |
| ESR, mm/hr, mean (SD) (n=348) | 10.0 (12.0) |
| CRP, mg/dL, mean (SD) (n=348) | 0.3 (1.1) |
| Patient global assessment (0-10), mean (SD) | 2.7 (1.4) |
| Physician global assessment (0-10), mean (SD) | 2.3 (1.3) |
| DAS28-ESR, mean (SD) (n=348) | 1.87 (0.83) |
| CDAI, mean (SD) | 5.8 (2.7) |
| SDAI, mean (SD) | 6.1 (3.0) |
| HAQ (0-3), mean (SD) | 0.7 (0.5) |

CDAI, clinical disease activity index; CRP, C-reactive protein; DAS, disease activity score; ESR, erythrocyte sedimentation rate; HAQ, health assessment questionnaire; SD, standard deviation; SDAI, simplified disease activity index

**Supplementary Table 2.** Clinical factors associated with achieving CDAI-remission

|  | Univariable analysis | | Multivariable analysis^*^ | |
| --- | --- | --- | --- | --- |
| Clinical factors | OR (95% CI) | *P* | OR (95% CI) | *P* |
| Age, year | 0.97 (0.95 to 0.996) | 0.021 | 0.99 (0.97 to 1.09) | 0.244 |
| Female sex | 0.96 (0.44 to 2.06) | 0.908 |  |  |
| Disease duration, year | 0.94 (0.90 to 0.99) | 0.010 | 0.96 (0.87 to 1.00) | 0.049 |
| BMI | 0.93 (0.85 to 1.01) | 0.086 | 0.94 (0.87 to 1.02) | 0.159 |
| Smoking history (vs. never-smoker) |  | 0.879 | ^†^ |  |
| Ex-smoker | 1.07 (0.40 to 2.90) |  |  |  |
| Current-smoker | 0.62 (0.08 to 4.48) |  |  |  |
| Seropositive RA (vs. seronegative RA) | 0.68 (0.25 to 1.90) | 0.465 | ^†^ |  |
| bDMARD-naïve | 1.33 (0.65 to 2.72) | 0.444 | ^†^ |  |
| SC tocilizumab (vs. IV tocilizumab) | 0.88 (0.42 to 1.61) | 0.573 | ^†^ |  |
| Baseline CDAI | 0.98 (0.95 to 1.01) | 0.207 | ^†^ |  |
| Baseline ESR (mm/hr) | 0.995 (0.98 to 1.01) | 0.441 | ^†^ |  |
| Baseline CRP (mg/dL) | 0.99 (0.90 to 1.08) | 0.773 | ^†^ |  |
| Baseline HAQ | 0.72 (0.50 to 1.02) | 0.061 | 0.79 (0.58 to 1.08) | 0.141 |
| Concomitant MTX use | 0.42 (0.24 to 0.74) | 0.003 | 0.57 (0.34 to 0.97) | 0.037 |
| Concomitant steroid use | 0.30 (0.16 to 0.55) | <0.001 | 0.47 (0.27 to 0.81) | 0.007 |
| CDAI measured at previous visit | 0.83 (0.75 to 0.93) | 0.001 | 0.83 (0.74 to 0.93) | 0.002 |

bDMARD, biologic disease-modifying anti-rheumatic drug; BMI, body mass index; CDAI, clinical disease activity index; CI, confidence interval; CRP, C-reactive protein; ESR, erythrocyte sedimentation rate; HAQ, health assessment questionnaire; HR, hazard ratio; IV, intravenous; MTX, methotrexate; RA, rheumatoid arthritis; SC, subcutaneous.

^*^, Including covariates with relevant association (P < 0.2) with the outcome in the univariable analysis

^†^, was not included in the multivariable model

**Supplementary Table 3.** Clinical factors associated with achieving DAS28-ESR remission

|  | Univariable analysis | | Multivariable analysis^*^ | |
| --- | --- | --- | --- | --- |
| Clinical factors | OR (95% CI) | *P* | OR (95% CI) | *P* |
| Age, year | 0.99 (0.98 to 1.01) | 0.354 | ^†^ |  |
| Female sex | 1.03 (0.59 to 1.87) | 0.924 | ^†^ |  |
| Disease duration, year | 0.99 (0.97 to 1.02) | 0.534 | ^†^ |  |
| BMI | 0.95 (0.89 to 1.02) | 0.160 | 0.95 (0.88 to 1.01) | 0.120 |
| Smoking history (vs. never-smoker) |  | 0.110 |  | 0.822 |
| Ex-smoker | 1.20 (0.52 to 2.74) |  | 0.97 (0.38 to 2.47) |  |
| Current-smoker | 0.30 (0.10 to 0.96) |  | 0.69 (0.21 to 2.22) |  |
| Seropositive RA (vs. seronegative RA) | 0.49 (0.22 to 1.07) | 0.072 | 0.61 (0.24 to 1.53) | 0.291 |
| bDMARD-naïve | 2.36 (1.46 to 3.81) | <0.001 | 2.30 (1.48 to 3.60) | <0.001 |
| SC tocilizumab (vs. IV tocilizumab) | 0.73 (0.45 to 1.20) | 0.219 | ^†^ |  |
| Baseline DAS28-ESR | 0.80 (0.67 to 0.96) | 0.014 | 0.94 (0.79 to 1.12) | 0.476 |
| Baseline HAQ | 0.81 (0.61 to 1.07) | 0.135 | 0.76 (0.59 to 0.99) | 0.045 |
| Concomitant MTX use | 0.91 (0.59 to 1.39) | 0.656 | ^†^ |  |
| Concomitant steroid use | 0.37 (0.25 to 0.55) | <0.001 | 0.44 (0.29 to 0.67) | <0.001 |
| DAS29-ESR measured at previous visit | 0.25 (0.19 to 0.35) | <0.001 | 0.27 (0.19 to 0.37) | <0.001 |

bDMARD, biologic disease-modifying anti-rheumatic drug; BMI, body mass index; DAS, disease activity score; CDAI, clinical disease activity index; CI, confidence interval; CRP, C-reactive protein; ESR, erythrocyte sedimentation rate; HAQ, health assessment questionnaire; HR, hazard ratio; IV, intravenous; MTX, methotrexate; RA, rheumatoid arthritis; SC, subcutaneous.

^*^, Including covariates with relevant association (P < 0.2) with the outcome in the univariable analysis

^†^, was not included in the multivariable model

**Supplementary Table 4.** Clinical factors associated with achieving DAS28-ESR deep remission

|  | Univariable analysis | | Multivariable analysis^*^ | |
| --- | --- | --- | --- | --- |
| Clinical factors | OR (95% CI) | *P* | OR (95% CI) | *P* |
| Age, year | 0.99 (0.98 to 1.01) | 0.483 | ^†^ |  |
| Female sex | 0.90 (0.51 to 1.57) | 0.703 | ^†^ |  |
| Disease duration, year | 1.002 (0.98 to 1.03) | 0.895 | ^†^ |  |
| BMI | 0.96 (0.90 to 1.01) | 0.129 | 0.94 (0.89 to 1.01) | 0.084 |
| Smoking history (vs. never-smoker) |  | 0.145 |  | 0.521 |
| Ex-smoker | 1.32 (0.62 to 2.84) |  | 1.20 (0.58 to 2.48) |  |
| Current-smoker | 0.30 (0.08 to 1.12) |  | 0.54 (0.16 to 1.83) |  |
| Seropositive RA (vs. seronegative RA) | 0.55 (0.29 to 1.06) | 0.072 | 0.61 (0.30 to 1.24) |  |
| bDMARD-naïve | 1.63 (1.03 to 2.59) | 0.027 | 1.58 (1.03 to 2.43) | 0.037 |
| SC tocilizumab (vs. IV tocilizumab) | 0.81 (0.52 to 1.26) | 0.353 | ^†^ |  |
| Baseline DAS28-ESR | 0.91 (0.79 to 1.05) | 0.202 | ^†^ |  |
| Baseline HAQ | 0.88 (0.69 to 1.11) | 0.279 | ^†^ |  |
| Concomitant MTX use | 0.89 (0.61 to 1.29) | 0.532 | ^†^ |  |
| Concomitant steroid use | 0.53 (0.37 to 0.75) | <0.001 | 0.69 (0.47 to 1.01) | 0.059 |
| DAS29-ESR measured at previous visit | 0.25 (0.19 to 0.34) | <0.001 | 0.27 (0.20 to 0.370 | <0.001 |

bDMARD, biologic disease-modifying anti-rheumatic drug; BMI, body mass index; DAS, disease activity score; CDAI, clinical disease activity index; CI, confidence interval; CRP, C-reactive protein; ESR, erythrocyte sedimentation rate; HAQ, health assessment questionnaire; HR, hazard ratio; IV, intravenous; MTX, methotrexate; RA, rheumatoid arthritis; SC, subcutaneous.

^*^, Including covariates with relevant association (P < 0.2) with the outcome in the univariable analysis

^†^, was not included in the multivariable model

**Supplementary Table 5.** Clinical factors associated with achieving SDAI remission

|  | Univariable analysis | | Multivariable analysis^*^ | |
| --- | --- | --- | --- | --- |
| Clinical factors | OR (95% CI) | *P* | OR (95% CI) | *P* |
| Age, year | 0.98 (0.96 to 0.99) | 0.010 | 0.99 (0.97 to 1.004) | 0.117 |
| Female sex | 1.34 (0.66 to 2.71) | 0.423 |  |  |
| Disease duration, year | 0.97 (0.94 to 1.002) | 0.063 | 0.98 (0.95 to 1.01) | 0.290 |
| BMI | 0.90 (0.84 to 0.97) | 0.006 | 0.93 (0.87 to 1.001) | 0.055 |
| Smoking history (vs. never-smoker) |  | 0.527 | ^†^ |  |
| Ex-smoker | 0.63 (0.24 to 1.67) |  |  |  |
| Current-smoker | 0.61 (0.15 to 2.50) |  |  |  |
| Seropositive RA (vs. seronegative RA) | 0.95 (0.34 to 2.64) | 0.919 | ^†^ |  |
| bDMARD-naïve | 1.33 (0.74 to 2.40) | 0.342 | ^†^ |  |
| SC tocilizumab (vs. IV tocilizumab) | 0.96 (0.54 to 1.68) | 0.872 | ^†^ |  |
| Baseline SDAI | 0.99 (0.96 to 1.01) | 0.203 | ^†^ |  |
| Baseline HAQ | 0.80 (0.60 to 1.07) | 0.125 | 0.88 (0.67 to 1.15) | 0.349 |
| Concomitant MTX use | 0.57 (0.34 to 0.93) | 0.024 | 0.67 (0.41 to 1.08) | 0.102 |
| Concomitant steroid use | 0.45 (0.28 to 0.74) | 0.001 | 0.68 (0.42 to 1.10) | 0.114 |
| SDAI measured at previous visit | 0.79 (0.72 to 0.87) | <0.001 | 0.80 (0.72 to 0.88) | <0.001 |

bDMARD, biologic disease-modifying anti-rheumatic drug; BMI, body mass index; CI, confidence interval; CRP, C-reactive protein; ESR, erythrocyte sedimentation rate; HAQ, health assessment questionnaire; HR, hazard ratio; IV, intravenous; MTX, methotrexate; RA, rheumatoid arthritis; SC, subcutaneous; SDAI, simplified disease activity index

^*^, Including covariates with relevant association (P < 0.2) with the outcome in the univariable analysis

^†^, was not included in the multivariable model

**Supplementary Table 6.** Clinical factors associated with achieving ACR/EULAR-remission

|  | Univariable analysis | | Multivariable analysis^*^ | |
| --- | --- | --- | --- | --- |
| Clinical factors | OR (95% CI) | *P* | OR (95% CI) | *P* |
| Age, year | 0.98 (0.96 to 0.996 | 0.018 | 0.98 (0.97 to 1.003) | 0.093 |
| Female sex | 1.26 (0.65 to 2.48) | 0.494 | ^†^ |  |
| Disease duration, year | 0.98 (0.95 to 1.004) | 0.093 | 0.99 (0.96 to 1.02) | 0.343 |
| BMI | 0.92 (0.85 to 0.99) | 0.024 | 0.93 (0.87 to 0.999) | 0.047 |
| Smoking history (vs. never-smoker) |  | 0.629 | ^†^ |  |
| Ex-smoker | 0.67 (0.25 to 1.78) |  |  |  |
| Current-smoker | 0.66 (0.16 to 2.74) |  |  |  |
| Seropositive RA (vs. seronegative RA) | 0.84 (0.34 to 2.05) | 0.703 | ^†^ |  |
| bDMARD-naïve | 1.51 (0.84 to 2.72) | 0.172 | 1.52 (0.89 to 2.60) | 0.122 |
| SC tocilizumab (vs. IV tocilizumab) | 1.13 (0.67 to 1.89) | 0.648 | ^†^ |  |
| Baseline CDAI | 0.99 (0.97 to 1.01) | 0.408 | ^†^ |  |
| Baseline ESR (mm/hr) | 0.99 (0.98 to 1.004) | 0.195 | 0.998 (0.99 to 1.01) | 0.680 |
| Baseline CRP (mg/dL) | 0.997 (0.93 to 1.07) | 0.930 | ^†^ |  |
| Baseline HAQ | 0.94 (0.71 to 1.26) | 0.686 | ^†^ |  |
| Concomitant MTX use | 0.71 (0.44 to 1.15) | 0.161 | 0.84 (0.53 to 1.34) | 0.467 |
| Concomitant steroid use | 0.49 (0.31 to 0.80) | 0.004 | 0.68 (0.43 to 1.08) | 0.105 |
| CDAI measured at previous visit | 0.80 (0.73 to 0.88) | <0.001 | 0.80 (0.72 to 0.89) | <0.001 |

bDMARD, biologic disease-modifying anti-rheumatic drug; BMI, body mass index; CDAI, clinical disease activity index; CI, confidence interval; CRP, C-reactive protein; ESR, erythrocyte sedimentation rate; HAQ, health assessment questionnaire; HR, hazard ratio; IV, intravenous; MTX, methotrexate; RA, rheumatoid arthritis; SC, subcutaneous.

^*^, Including covariates with relevant association (P < 0.2) with the outcome in the univariable analysis

^†^, was not included in the multivariable model

**Supplementary Figure 3.** Longitudinal disease activity in the study population

**Supplementary Figure 4.** Estimated HAQ-DI according to the occurrence of CDAI-low disease activity (LDA) in a one-year interval. Intervals with loss of CDAI-LDA was significantly associated with higher HAQ-DI. Error bar indicates 95% confidence interval.

**Supplementary Figure 5** Estimated HAQ-DI over time between the two groups. Error bar indicates 95% confidence interval.

**Supplementary Table 7.** Effect of tapering tocilizumab on loss of CDAI-LDA in the multivariable GEE model including all clinically relevant factors

|  | Univariable analysis | | Multivariable analysis | |
| --- | --- | --- | --- | --- |
| Clinical factors | OR (95% CI) | *P* | OR (95% CI) | *P* |
| Age, year | 1.01 (0.99 to 1.03) | 0.472 | 0.996 (0.98 to 1.02) | 0.721 |
| Female sex | 0.83 (0.42 to 1.64) | 0.592 | 0.995 (0.31 to 3.16) | 0.993 |
| Disease duration, year | 1.02 (0.99 to 1.05) | 0.215 | 1.02 (0.99 to 1.05) | 0.283 |
| BMI | 1.09 (1.002 to 1.18) | 0.046 | 1.10 (1.01 to 1.20) | 0.029 |
| Smoking history (vs. never-smoker) |  | 0.057 |  | 0.630 |
| Ex-smoker | 0.87 (0.34 to 2.25) |  | 1.38 (0.32 to 5.97) |  |
| Current-smoker | 3.32 (1.22 to 9.09) |  | 2.06 (0.47 to 9.05) |  |
| Seropositive RA (vs. seronegative RA) | 1.33 (0.54 to 3.26) | 0.532 | 1.13 (0.50 to 2.56) | 0.779 |
| bDMARD-naïve | 0.56 (0.32 to 0.97) | 0.039 | 0.56 (0.31 to 0.98) | 0.044 |
| SC tocilizumab (vs. IV tocilizumab) | 2.03 (1.19 to 3.49) | 0.010 | 3.31 (1.65 to 6.62) | 0.001 |
| Baseline CDAI | 1.01 (0.98 to 1.03) | 0.646 | 0.99 (0.96 to 1.01) | 0.266 |
| Baseline ESR (mm/hr) | 1.01 (1.001 to 1.02) | 0.036 | 1.01 (1.001 to 1.02) | 0.033 |
| Baseline HAQ | 1.39 (1.01 to 1.91) | 0.043 | 1.23 (0.85 to 1.79) | 0.272 |
| Concomitant MTX use | 1.15 (0.67 to 2.00) | 0.614 | 1.11 (0.63 to 1.94) | 0.718 |
| Concomitant steroid use | 2.18 (1.28 to 3.70) | 0.004 | 1.91 (1.10 to 3.33) | 0.023 |
| CDAI measured at previous visit | 1.18 (1.11 to 1.27) | <0.001 | 1.11 (1.04 to 1.19) | 0.003 |
| Tapering group | 1.02 (0.67 to 1.55) | 0.929 | 1.75 (1.01 to 3.03) | 0.045 |

bDMARD, biologic disease-modifying anti-rheumatic drug; BMI, body mass index; CDAI, clinical disease activity index; CI, confidence interval; CRP, C-reactive protein; ESR, erythrocyte sedimentation rate; HAQ, health assessment questionnaire; HR, hazard ratio; IV, intravenous; MTX, methotrexate; RA, rheumatoid arthritis; SC, subcutaneous.

**Supplementary Table 8.** Effect of tapering tocilizumab on loss of CDAI-LDA in the multivariable GEE model with ‘exchangeable’ correlation matrix

|  | Univariable analysis | | Multivariable analysis^*^ | |
| --- | --- | --- | --- | --- |
| Clinical factors | OR (95% CI) | *P* | OR (95% CI) | *P* |
| Age, year | 1.01 (0.99 to 1.03) | 0.552 | ^†^ |  |
| Female sex | 0.79 (0.39 to 1.61) | 0.524 | ^†^ |  |
| Disease duration, year | 1.02 (0.99 to 1.05) | 0.208 | ^†^ |  |
| BMI | 1.09 (1.003 to 1.18) | 0.043 | 1.01 (1.00 to 1.02) | 0.058 |
| Smoking history (vs. never-smoker) |  | 0.055 |  | 0.459 |
| Ex-smoker | 0.91 (0.32 to 2.62) |  | 1.39 (0.48 to 3.99) |  |
| Current-smoker | 3.46 (1.25 to 9.58) |  | 1.96 (0.62 to 6.19) |  |
| Seropositive RA (vs. seronegative RA) | 1.07 (0.39 to 2.97) | 0.902 | ^†^ |  |
| bDMARD-naïve | 0.58 (0.33 to 1.01) | 0.054 | 0.53 (0.30 to 0.92) | 0.024 |
| SC tocilizumab (vs. IV tocilizumab) | 2.03 (1.18 to 3.49) | 0.010 | 3.13 (1.58 to 6.21) | 0.001 |
| Baseline CDAI | 1.01 (0.98 to 1.03) | 0.683 | ^†^ |  |
| Baseline ESR (mm/hr) | 1.01 (1.001 to 1.02) | 0.026 | 1.01 (1.00 to 1.02) | 0.058 |
| Baseline CRP (mg/dL) | 1.04 (0.97 to 1.11) | 0.230 | ^†^ |  |
| Baseline HAQ | 1.40 (1.02 to 1.92) | 0.037 | 1.12 (0.79 to 1.59) | 0.535 |
| Concomitant MTX use | 1.31 (0.77 to 2.23) | 0.325 | ^†^ |  |
| Concomitant steroid use | 2.07 (1.20 to 3.56) | 0.009 | 1.87 (1.08 to 3.26) | 0.026 |
| CDAI measured at previous visit | 1.16 (1.09 to 1.24) | <0.001 | 1.13 (1.06 to 1.21) | <0.001 |
| Tapering group | 1.04 (0.68 to 1.59) | 0.866 | 1.79 (1.01 to 3.16) | 0.045 |

bDMARD, biologic disease-modifying anti-rheumatic drug; BMI, body mass index; CDAI, clinical disease activity index; CI, confidence interval; CRP, C-reactive protein; ESR, erythrocyte sedimentation rate; HAQ, health assessment questionnaire; HR, hazard ratio; IV, intravenous; MTX, methotrexate; RA, rheumatoid arthritis; SC, subcutaneous.

^*^, Including covariates with relevant association (P < 0.2) with the outcome in the univariable analysis

^†^, was not included in the multivariable model
